# PDMAC: A Priority-Based Enhanced TDMA Protocol for Warning Message Dissemination in VANETs

**DOI:** 10.3390/s20010045

**Published:** 2019-12-19

**Authors:** Ghulam Abbas, Ziaul Haq Abbas, Shahab Haider, Thar Baker, Saadi Boudjit, Fazal Muhammad

**Affiliations:** 1 Faculty of Computer Science and Engineering, GIK Institute of Engineering Sciences and Technology, Topi 23640, Pakistan; abbasg@giki.edu.pk; 2 Faculty of Electrical Engineering, GIK Institute of Engineering Sciences and Technology, Topi 23640, Pakistan; ziaul.h.abbas@giki.edu.pk; 3 Telecommunications and Networking (TeleCoN) Research Lab, GIK Institute of Engineering Sciences and Technology, Topi 23640, Pakistan; shahab@giki.edu.pk; 4 Department of Computer Science, Faculty of Engineering and Technology, Liverpool John Moores University, Liverpool L3 3AF, UK; 5 Laboratoire de Traitement et Transport de l’Information Lab (L2TI), Institut Galilée, University of Paris 13, 99 Av J-Baptiste Clément, 93430 Villetaneuse, France; boudjit@univ-paris13.fr; 6 Department of Electrical Engineering, City University of Science and Information Technology, Peshawar 25000, Pakistan; fazal.muhammad@cusit.edu.pk

**Keywords:** clock synchronization, media access control protocol, time-division multiple access, vehicular ad hoc networks, warning message dissemination

## Abstract

Vehicular ad hoc networks (VANETs) are the key enabling technology for intelligent transportation systems. Carrier-sense multiple access with collision avoidance (CSMA/CA) is the de facto media access standard for inter-vehicular communications, but its performance degrades in high-density networks. Time-division multiple access (TDMA)-based protocols fill this gap to a certain extent, but encounter inefficient clock synchronization and lack of prioritized message delivery. Therefore, we propose a priority-based direction-aware media access control (PDMAC) as a novel protocol for intra-cluster and inter-cluster clock synchronization. Furthermore, PDMAC pioneers a three-tier priority assignment technique to enhance warning messages delivery by taking into account the direction component, message type, and severity level on each tier. Analytical and simulation results validate the improved performance of PDMAC in terms of clock synchronization, channel utilization, message loss rate, end-to-end delays, and network throughput, as compared with eminent VANET MAC protocols.

## 1. Introduction

### 1.1. Motivation and Objectives

Vehicular ad hoc networks (VANETs) enable communication among high-speed vehicles (hereafter, nodes) in an intelligent transportation system (ITS) [1]. ITSs have several applications (e.g., smart cities, infotainment, route and travel time estimation, and accident prevention) [2]. However, accident prevention attracts more attention due to the over 1.25 million deaths and 20–50 million critical injuries caused by road accidents each year around the globe [3].

To evade road accidents, cooperative collision avoidance (CCA) schemes compute collision probabilities at regular intervals among nodes and encapsulate them in warning messages along with appropriate preventive measures [4]. These messages are transmitted either through a vehicle-to-vehicle (V2V) or a vehicle-to-infrastructure (V2I) communication model. A V2V model establishes communication among nodes directly, while a V2I model employs road-side units (RSUs) for message transmission and has increased deployment and maintenance costs [5,6]. In this regard, cluster-based approaches effectively manage the nodes and prevent broadcast floods by restricting the broadcast domain to the individual clusters, thereby minimizing the communication overhead [1,7].

Besides the identification of a possible collision among nodes, reliable and in-time delivery of warning messages is also critical in CCA schemes [8]. This is because preventive measures can only be effective if the nodes find ample time to take these measures. Issues related to the warning message dissemination are addressed both at network and media access control (MAC) layers. Network layer protocols seek to find the best route to reach a certain destination node in a multi-hop environment [5,9], whereas protocols at the MAC layer ensure message delivery over a single link while preventing channel access collisions. This paper focuses on the MAC layer, where communications over a shared medium remain critical.

Toward that end, carrier-sense multiple access with collision avoidance (CSMA/CA) is considered as the de facto media access standard for inter-vehicular communication at the MAC layer [10]. However, it experiences performance degradation in the face of high node density [11]. This demands efficient channel utilization and message delivery at the MAC layer. The protocols that adopt time-division multiple access (TDMA) fill this gap to a certain extent. However, as a result of frequent topological changes in VANETs due to high-speed nodes moving in opposite directions, TDMA-based protocols also experience performance degradation in terms of increased end-to-end delays and message losses, which adversely impact the network throughput. One of the major reasons for this is the lack of consideration of the direction component during the relay selection process on bi-directional highways.

Moreover, clock synchronization is another issue in TDMA-based protocols [12,13]. The existing literature focuses only on the intra-cluster clock synchronization. However, it is not necessary that a path only comprises nodes from a single cluster; rather, relay services of nodes from other clusters are also frequently acquired in VANETs. In such cases, time slot reservations become challenging for messages generated by nodes from different clusters. This is because local clocks of nodes in different clusters may bear different clock times, which result in inefficient time slot reservation, utilization, and release, thus producing channel collisions at a large scale. To overcome this issue, the need for inter-cluster clock synchronization becomes critical in addition to intra-cluster clock synchronization.

Furthermore, since warning messages are time-sensitive, CCA schemes require the prioritized delivery of warning messages. A promising approach in this regard is to assign higher priority to warning messages over non-warning messages (A non-warning message refers to any message other than the warning message, which can be used to communicate route identification, traffic density information, etc. [14,15].) (see, e.g., [16]). However, treating all warning messages with equal priority limits the performance of this approach because the probability of collision among nodes may not remain the same all the time. This adversely affects the delivery of warning messages with high probability of collision, especially in dense networks.

The objective of this paper is to address the aforementioned challenges of TDMA-based MAC protocols by presenting a novel protocol that enables reliable and in-time delivery of time-critical warning messages in VANETs. This will provide ample time for nodes to implement preventive measures, thereby reducing the number of road accidents.

### 1.2. Novelty and Contributions

We propose a priority-based direction-aware media access control (PDMAC) protocol which makes the following contributions:

1. PDMAC introduces *inter-cluster clock synchronization* in addition to intra-cluster clock synchronization, using a V2V model on bi-directional highways. We show that this leads to fast clock synchronization with reduced communication overhead and improved channel utilization.
2. PDMAC introduces a *three-tier priority assignment* technique to ensure prioritized delivery of time-sensitive warning messages, as follows.
  - The first tier takes into account the direction of nodes for selecting relays. This helps to reduce the message loss rate and end-to-end delays, and improves the network throughput.
  - The second tier prioritizes the time-critical warning messages over non-warning messages.
  - The third tier further prioritizes warning message on the basis of different severity levels, where a severity level is proportional to the probability of collision among nodes. Such a prioritized transmission helps to enhance the delivery ratio of warning messages, thereby providing better collision avoidance among nodes.

To the best of our knowledge, PDMAC is a pioneering approach to employ a three-tier priority assignment and exploit inter-cluster clock synchronization besides intra-cluster clock synchronization.

### 1.3. Paper Organization

The rest of the paper is organized as follows. Section 2 reviews state-of-the-art MAC protocols in VANETs. Section 3 details the proposed PDMAC protocol. Section 4 evaluates the performance of the proposed protocol. Finally, Section 5 concludes the paper with future research directions. Table 1 lists the notations used in this paper.

## 2. Related Work

In dynamic networks like VANETS, TDMA-based protocols perform better than CSMA/CA with respect to message delivery rate [11]. However, inefficient clock synchronization limits the performance of TDMA-based protocols [13]. In this regard, the authors in [17] present an intra-cluster clock synchronization technique, referred to as the CSRDS protocol in this paper. CSRDS introduces an approximate agreement approach to avoid Global Positioning System (GPS)-based clock synchronization. Similar intra-cluster clock synchronization techniques have also been presented [12,18,19]. However, unless the local clocks of all the nodes in a network are synchronized to a commonly shared clock through inter-cluster clock synchronization, the time slot reservation, its utilization, and release on successful or unsuccessful delivery of messages always remain inefficient. Distributed multi-channel MAC (DMCMAC) [14] synchronizes its local clock with the GPS. However, in such an approach the clocks remain unsynchronized when GPS is not available (e.g., inside tunnels). Another clock synchronization protocol proposed in [20] employs epoch time to evaluate and synchronize the local current round-time. However, time slot shifting correction is a major limitation of this work.

The lack of prioritized message transmission is another issue in TDMA-based protocols. Prediction-based TDMA MAC (PTMAC) [21] detects packet collisions on the channels. Similarly, optimal cooperative ad hoc-MAC (OCA-MAC) [22] considers relay and destination nodes with available time slots to compute an optimal path. Moreover, VANET adaptive TDMA-MAC (VAT-MAC) [23] optimizes frame length by predicting the number of nodes in the network. Furthermore, the authors in [24,25,26] allocate disjoint sets of time slots that minimize the channel collisions. However, the availability of such disjoint sets at all times is unrealistic. In addition, the authors in [27,28] propose hybrid protocols that combine the functionality of CSMA with TDMA to enhance message delivery ratio, but face performance degradation as the network density increases.

Similarly, TDMA-aware routing protocol for multi-hop communication (TRPM) [29] enables reliable long-distance communication. It selects a relay based on a delay-tolerant MAC (DTMAC) scheduling scheme, which exhibits the problem of channel access collisions [30]. Furthermore, the work in [31] presents a novel software-defined network (SDN)-based protocol for warning messages dissemination and introduces the concept of open flow switch. However, the selection of an SDN controller in VANETs is a challenging task due to its highly dynamic topology. The authors in [32,33] propose adaptive techniques for time slot reservation, which improve channel access. However, the warning messages delivery ratio deteriorates due to non-prioritized slot allocation. Similarly, cluster-based MAC (CB-MAC) [34] avoids the use of request to send (RTS) and clear to send (CTS) messages in order to minimize the warning messages communication overhead. However, the lack of such essential handshake messages may result in access collisions at a large scale. Moreover, mobility-aware collision avoidance MAC (MoMAC) [35] proposes an even distribution of time slots among road lanes, which is unrealistic and may result in time slot wastage in real traffic.

The work in [36] presents the triggered control channel interval (CCHI) multi-channel MAC (TCM-MAC) protocol, which allocates variable time slots to messages for their transmission. The authors in [37] propose the use of variable transmission power to enhance message delivery ratio. RSU-assisted multi-channel protocol [38] employs RSUs for time interval optimization and message tracking. However, the deployment and maintenance costs of RSUs is a limitation. The work in [14] proposes an adaptive DMCMAC protocol, which divides the number of time slots on the frames in accordance with the number of messages to be transmitted on the network in order to enhance channel access. However, treating the warning and non-warning messages with equal priority degrades the performance of this protocol, especially when the number of non-warning messages is higher than that of the warning messages. The work in [39] takes the congestion level into account in order to prioritize messages. However, this approach also treats both warning and non-warning messages with the same priority. The authors in [16,40,41] propose TDMA-based MAC protocols that prioritize warning messages over non-warning messages. This improves the delivery rate of time-sensitive warning messages to a certain extent. However, these protocols do not differentiate between warning messages of different severity levels. Thus, there is a need to further prioritize warning messages based on the severity level, which can be determined from the probability of collision among nodes.

From the literature survey, we found that the existing TDMA-based MAC protocols provide timely and reliable delivery of warning messages in VANETs, mainly by using intra-cluster clock synchronization approaches. However, it is not necessary that a path only comprises nodes from a single cluster, as relay services of nodes from other clusters are also frequently acquired in VANETs. Nevertheless, the existing approaches lack inter-cluster clock synchronization, and thus these approaches fail when time slot reservations are carried out for nodes belonging to different clusters. Moreover, the current literature on MAC protocols for VANETs does not take into account the direction component of nodes, and thus cannot cater to bi-directional highways where high-speed nodes move in opposite directions, causing frequent topological changes. Furthermore, the current body of literature provides MAC protocols that are capable of prioritizing warning messages over non-warning messages. However, these protocols lack the ability to differentiate between warning messages of different severity. To address the aforementioned challenges, we propose a novel solution called PDMAC, which is described in the following section.

## 3. Priority-Based Direction-Aware Media Access Control (PDMAC) Protocol

**Figure 1:**
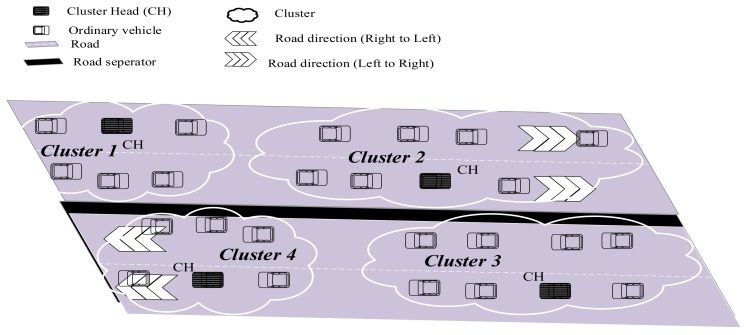
A bi-directional highway traffic scenario.

**Figure 2:**
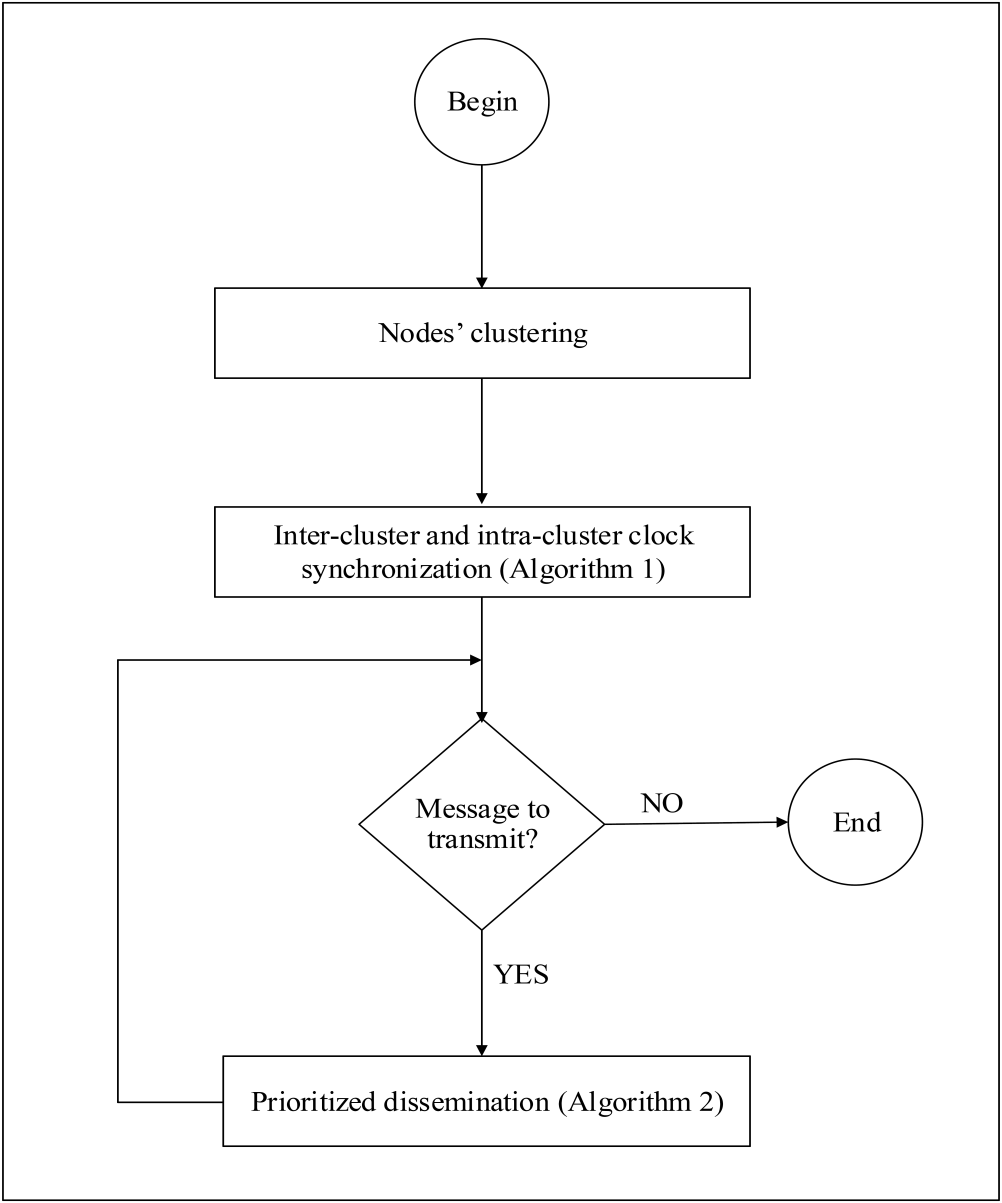
Overview of the proposed priority-based direction-aware media access control (PDMAC) protocol.

This section presents our proposed PDMAC protocol for V2V warning message dissemination on bi-directional highways, as depicted in Figure 1. The methodology of PDMAC is to start with nodes’ clustering to enable enhanced node manageability and to restrict the broadcast domains (see Section 3.1). This is followed by the clock synchronization of nodes, which is critical for time slot reservation (see Section 3.2). Here, we introduce a local clock synchronization technique that is composed of two phases, namely, inter-cluster and intra-cluster clock synchronizations. We then present the proposed three-tier priority assignment technique to enhance the delivery rate of time-sensitive warning messages (see Section 3.3). Finally, we present the time complexity of PDMAC (see Section 3.4). An overview of the proposed solution is presented in Figure 2.

### 3.1. Node Clustering

In TDMA-based MAC protocols, clock synchronization is one of the most important factors for message transmission. Since each node uses a specific time slot (${ℸ}_{s}$) to transmit its message, it becomes inevitable to synchronize the local clocks of all nodes on the network. In this regard, cluster-based approaches are promising, as the limited broadcast domain due to clustering reduces the communication overhead and prevents broadcast floods to a significant extent [1,7,18,19]. In all such approaches, clustering is performed as soon as a node joins the highway. Once a node is a member or cluster head (CH) of a cluster, it can transmit messages. This allows nodes to timely transmit warning messages in critical situations without having to perform clustering each time before sending a message. Thus, clustering is not performed just before a critical event, such as an incipient collision, and it does not have any adverse effect in critical situations. Rather, enhanced node manageability results in improved performance due to clustering [4].

PDMAC clusters nodes on the network by using a VANET-specific variant of the *k*-medoids algorithm, proposed in our previous work [4]. However, in [4] clustering is performed at the application layer with the aim of avoiding road accidents, whereas in this paper we use clustering for warning message dissemination at the MAC layer. The process of clustering in PDMAC initiates as soon as a node enters the highway. Here, we have two types of nodes: cluster heads (CHs) and ordinary vehicles (OVs). A CH manages a cluster and keeps the record of all its member nodes. Conversely, an OV represents any node other than the CH. However, the status of an OV changes to member node as soon as it joins a certain cluster.

### 3.2. Clock Synchronization

On successful completion of node clustering, the process of clock synchronization is initiated. The local clocks of nodes are synchronized to a commonly shared clock in the following two phases.

#### 3.2.1. Inter-Cluster Clock Synchronization

To synchronize the local clocks of all nodes on the network, PDMAC introduces a single-bit field—namely, the node’s timer validation bit (validate_timer)—in the message header. This field indicates whether or not the timer is synchronized with the other network nodes. If validate_timer = 1, the node’s timer is considered to be synchronized and, hence, valid. Conversely, if validate_timer = 0, the clock is required to be synchronized and validate_timer remains invalid to all other nodes on the network. PDMAC keeps the default validate_timer as 0 to make clock synchronization mandatory for all the nodes on their entry to the highway.

The clock synchronization process (Algorithm 1) starts after the completion of the clustering process, that is, when all nodes in the network are clustered and a CH for each cluster is elected using the *k*-medoids algorithm (see Section 3.1). The set of all CHs in the network, which are elected by means of the *k*-medoids algorithm, is denoted as $\bar{CH}$. The first phase of Algorithm 1 synchronizes the clocks of $\bar{CH}$. For this *inter-cluster* clock synchronization, a CH is arbitrarily chosen from $\bar{CH}$, and is denoted as $CH_{B}$. The rest of the CHs then synchronize their local clocks with the commonly shared clock of $CH_{B}$ as follows.

$CH_{B}$ broadcasts a CH’s clock synchronization message ($Sync_{CH}$), which is acknowledged by all reachable CHs. It must be noted that OVs do not update their clocks on reception of $Sync_{CH}$ and are only used as relay nodes to forward this message to the CHs. The validate_timer for $CH_{B}$ is set to 1, so that all other CHs can synchronize their timer to this randomly chosen CH. A CH with an unsynchronized timer changes its local time to that of $CH_{B}$. As soon as a CH synchronizes its local clock, its validate_timer is set to 1 and in this way all the CHs are synchronized to a common local clock. Here, it is not mandatory for each new CH to synchronize its clock with $CH_{B}$ only. Any CH with a validated clock can validate other CHs as soon as it receives a request for clock synchronization.

#### 3.2.2. Intra-Cluster Clock Synchronization

On completion of the first phase, the process of intra-cluster local clock synchronization initiates. Here, each CH multicasts a member clock synchronization message ($Sync_{MEM}$) to its member nodes for the communication of its local clock time. If the validated_timer of a node is 0, the member updates its local clock time ($T_{C_{i}}$) with respect to its corresponding CH and flips its validated_timer to 1, which indicates that the node’s timer is now synchronized. However, in the case of a node with validated_timer = 1, no further synchronization action is required. Moreover, a validated node does not need to synchronize its timer again if it is elected as a CH in the future.

**Figure 3:**
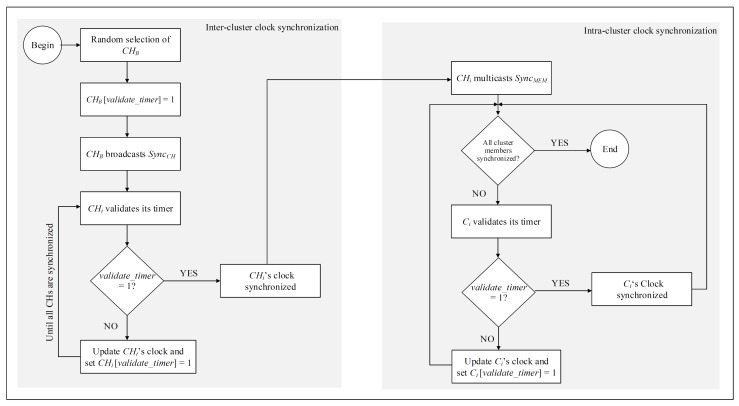
Procedural flowchart of the clock synchronization algorithm.

The proposed inter-cluster and intra-cluster clock synchronization technique is presented in Algorithm 1. The algorithm takes *N*, $\bar{CH}$, and *C* as input, where *N* represents the set of all nodes, $\bar{CH}$ is the set of cluster heads, and *C* represents the set of member nodes in each cluster. The output of this algorithm includes synchronized local clocks of all the nodes on the network. After clock synchronization, nodes can perform prioritized message dissemination, the procedure for which is detailed in the following section. Figure 3 presents the procedural flowchart of Algorithm 1.

### 3.3. Prioritized Warning Message Dissemination

In PDMAC, when a source node (*S*) intends to transmit a warning message (*W*) to a certain destination (*D*) and these nodes lie within communication range of each other, *S* disseminates the message straightaway by reserving all available time slots in its frame to itself. Otherwise, *S* requests its neighboring nodes to provide relay services. Neighbors include all the nodes that lie within the communication range of *S*, and a suitable intermediary node (The terms intermediary node and forwarder are used interchangeably in this paper to refer to a relay node.) among the neighbors is selected to relay the message from *S* to *D*. To find a suitable relay node, *S* broadcasts a request message (REQ) to its neighbors. An REQ message includes the following fields: Source Identity (SID), Destination Identity (DID), Source Direction Information (SDI), Destination Direction Information (DDI), Message Type (SN), and warning message Severity Level (SL). Each neighbor responds to *S* with an acknowledgment message (ack) that includes Relay node Identity (RID), Relay node Direction Information (RDI), set of free time slots ($\alpha_{f}$), and time slot to be assigned (${ℸ}_{s}$). *S* selects the best forwarder ($B_{f}$) and sends a response message (RES) to it only. An RES message includes the best forwarder Identity ($B_{f}\text{\_}ID$) and relay services acceptance/rejection decision (dec). To accept the relay services of a node, dec is set to 1. Furthermore, REQ and ack use the control channel (CCH), whereas the RES utilizes the service channels (SCHs). PDMAC implements a three-tier priority assignment process to enhance the delivery of warning messages, which is detailed in the following subsections.

**Algorithm 1** Clock synchronization.**Input:***N*, $\bar{CH}$, and *C* **Output:** Synchronized time for all CHs and member nodes
**Begin:**
**Set**
*N*[validate_timer] **as** OFF 
     **Inter-cluster Clock Synchronization:**
            $CH_{B}$⟵ Rand($\bar{CH}$)            **Set**
$CH_{B}$[validate_timer] **as** ON            $CH_{B}$ broadcasts $Sync_{CH}$               **For**
*i* = 1 **To** sizeof($\bar{CH}$)                      **If**
$CH_{i}$[validate_timer] = OFF **Then**                          $T_{CH_{i}}$⟵$T_{CH_{B}}$                          **Set**
$CH_{i}$[validate_timer] **as** ON                      **Else**                          No clock synchronization is required                      **End If**               **End For** 
     **Intra-cluster Clock Synchronization:**        **For**
*j* = 1 **To** sizeof($\bar{CH}$)            $CH_{j}$ multicasts $Sync_{MEM}$ to its member nodes               **For**
*i* = 1 **To** sizeof(*C*)                   $C_{i}$ sends $ack_{i}$ to $CH_{j}$                      **If**
$C_{i}$[validate_timer] = OFF **Then**                          $T_{C_{i}}$⟵$T_{CH_{j}}$                          **Set**
$C_{i}$[validate_timer] **as** ON                      **Else**                          No clock synchronization is required                      **End If**               **End For**
        **End For** 
**End**


#### 3.3.1. Tier 1—Direction-Based Relay Selection

The high-speed mobility of nodes in opposite directions on highways causes frequent topological changes in the form of route breakages and reconstructions, which result in network partitions [42]. Therefore, it becomes necessary to consider the movement direction of nodes during the selection of relays. Toward this end, PDMAC first computes the L1-norm distance (μ) between each of the possible relays ($R_{i}$) and destination node (*D*), using the technique proposed in [5]. The protocol then considers the direction component (κ) by using the Hamming distance function (H(.)) and the technique proposed in [5]. The outcome of H(.) is 1 if $R_{i}$ is moving in the direction of *D*, and it will be 0 if the direction of $R_{i}$ is opposite to *D*. The final distance (δ) between each $R_{i}$ and *D* is computed in terms of μ and κ. In the case where H(S,D) = 1, and *D* is in front of *S*, δ is computed as

(1) $$\delta_{i}=\frac{\mu_{i}}{\kappa_{i}}.$$

Furthermore, when *S* is in front of *D* having H(S,D) = 1, δ is computed as

(2) $$\delta_{i}=\mu_{i}\kappa_{i}.$$

Similarly, for H(S,D) = 0 with *S* and *D* moving towards each other, (1) is used to compute δ. Alternatively, if H(S,D) = 0 and *S* and *D* are moving away from each other, (2) is used. Finally, a minimum function (Min(.)) identifies the $B_{f}$ among the available set of intermediary relay nodes (*R*), which assigns highest priority to the relay closest in distance to *D* and having direction towards it. On successful completion of the aforementioned process, PDMAC updates the relay identification field (Next_hop) of the message header by adding the Node Identity (NID) of the selected $B_{f}$.

#### 3.3.2. Tier 2—Priority on the Basis of Message Type

Unlike non-warning messages, warning messages are time-critical and delays during their transmission may result in collisions among nodes at a large scale [12]. So, Tier-2 priority assignment in the proposed PDMAC protocol is to differentiate between warning and non-warning messages and to assign a higher priority to warning messages. PDMAC introduces a single-bit field in the message header, namely, message type (SN), to identify the type of a certain message. For warning messages, SN is set to 1, whereas SN remains 0 in case of non-warning messages.

The proposed SN-based Tier-2 priority assignment seeks to improve the delivery of warning messages to a certain extent. However, further prioritization of warning messages is essential because assigning equal priority to warning messages of low- as well as high-severity events degrades the performance of a CCA scheme, as discussed in Section 2. The severity levels of different critical events may be different. Thus, a warning message of a higher-severity event (e.g., an incipient road accident) should receive a higher priority. To address this issue, we propose a Tier-3 priority assignment in the following subsection which further prioritizes the warning messages based on their severity levels.

#### 3.3.3. Tier 3—Priority on the Basis of Severity Levels

The third tier of priority assignment in PDMAC is to determine the priority of warning messages based on the severity of a critical event (e.g., an incipient collision among nodes). In this tier, warning messages are differentiated from each other based on their severity levels measured on the basis of the probability of collision among nodes. However, computing the collision probability is generally the task of an application layer protocol. In this regard, our previous work [4] proposes a technique to compute the collision probability based on relative speeds, relative distances, and the direction of nodes. It then determines the safe speed for nodes to evade a collision. The collision probability along with the safe speed is communicated to the rear node, which then adopts the safe speed to avoid the collision. However, since this paper concerns the MAC layer, for the sake of determining the priority of warning messages on the basis of severity level, we assume that the collision probability is available to PDMAC from the application layer protocol. PDMAC employs this collision probability to determine the Severity Level (SL) of a certain warning message according to Table 2.

Warning messages are classified into three levels, namely, $SL_{0}$, $SL_{1}$, and $SL_{2}$, as shown in Table 2. Here, $SL_{0}$ represents a warning message with the lowest collision probability, whereas $SL_{2}$ refers to the one with the highest collision probability. Moreover, for non-warning messages (NWs) with SN=0, the probability of collision always remains 0. This implements the third-tier priority in ${ℸ}_{s}$ reservation, for which PDMAC introduces a 2-bit SL field in the message header.

In case of a warning message that belongs to the $SL_{0}$ category, *S* waits for a free ${ℸ}_{s}$, which keeps this type of warning message on the lowest priority. $SL_{1}$ increases the priority level, such that *S* can request to release a ${ℸ}_{s}$ occupied by a non-warning message or a warning message of lower priority. If none of these options are available, then $SL_{1}$ warning messages also wait for a ${ℸ}_{s}$ to become available, as it is not obligatory upon other nodes to respond and release their occupied ${ℸ}_{s}$. Finally, if an $SL_{2}$ level warning message does not find any free ${ℸ}_{s}$, it is mandatory for non-warning messages and lower-priority warning messages to release their allotted ${ℸ}_{s}$ for it. This ensures reliable and in-time delivery of highly critical warning messages. It is worth mentioning here that an $SL_{2}$ level warning message also behaves like $SL_{0}$ or $SL_{1}$ messages in a case where all ${ℸ}_{s}$ are occupied by the warning messages of similar Tier-3 priority, which is extremely rare.

**Figure 4:**
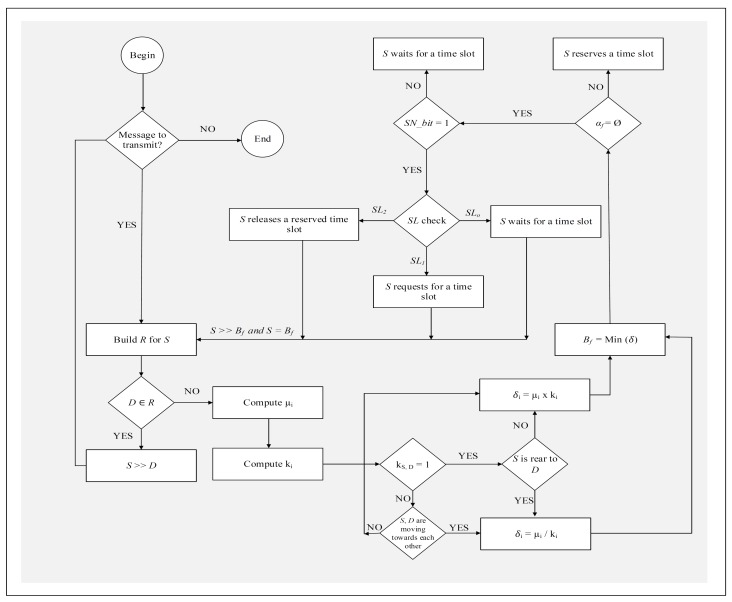
Procedural flowchart of the prioritized warning message dissemination algorithm.

Our proposed three-tier priority assignment technique is presented in Algorithm 2. The algorithm takes *S*, *D*, *R*, $\alpha_{f}$, and *W* as inputs, where *R* represents the set of intermediary relay nodes, $\alpha_{f}$ is the set of free ${ℸ}_{s}$, and *W* refers to a warning message. The output of the algorithm includes the selection of $B_{f}$ and the reservation of ${ℸ}_{s}$ to transmit *W*. Figure 4 presents the flowchart of the three-tier priority assignment process of Algorithm 2.

**Algorithm 2** Prioritized warning message dissemination.**Input:***S*, *D*, *R*, $\alpha_{f}$, and *W* **Output:**$B_{f}$ selection and time slot reservation for warning message dissemination 
**Begin:**
**Repeat**
  **If**
*D*∈*R*
**Then**     *S*>>*D*
  **Else**
     **For**
*i* = 1 **To** sizeof(*R*)
        $\mu_{i}$⟵$|D_{x}-R_{i_{x}}|$ + $|D_{y}-R_{i_{y}}|$
        $\kappa_{i}$⟵$H(R_{i},D)$            **If**
H(S,D) = 1 **Then**               **If**
*S* = Rear & *D* = Front node **Then**                   $\delta_{i}$⟵$\frac{\mu_{i}}{\kappa_{i}}$               **Else**                   $\delta_{i}$⟵$\mu_{i}$$\kappa_{i}$               **End If**            **Else**               **If**
*S*, *D* move towards each other **Then**                   $\delta_{i}$⟵$\frac{\mu_{i}}{\kappa_{i}}$               **Else**                   $\delta_{i}$⟵$\mu_{i}$$\kappa_{i}$               **End If**            **End If**
     **End For** 
     $B_{f}$⟵ Min(δ) 
     **If**
$\alpha_{f}$ = ϕ
**Then**        **If**
*W*[SN_bit] = 1            *ℓ*⟵*W*[severity_bits]            **Switch**(*ℓ*)               **Case:** 00                   *S* waits for a free ${ℸ}_{s}$               **Case:** 01                   *S* requests to release a ${ℸ}_{s}$               **Case:** 10                   *S* releases ${ℸ}_{s}$ already reserved by                   a non-warning or a lower priority warning                   message.            **End Switch**        **Else**            *S* waits for a free ${ℸ}_{s}$        **End If**
     **Else**
        *S* reserves a ${ℸ}_{s}$ from $\alpha_{f}$
     **End If**
        *S*>>$B_{f}$
        *S*⟵$B_{f}$
  **End If**
**Until**$\;\;B_{f}$ = *D*
**End**


### 3.4. Time Complexity

Time complexity refers to the number of steps carried out for the dissemination of a message from *S* to *D*. In our proposed PDMAC protocol, Algorithm 1 is composed of two major sections, where the first is responsible for inter-cluster clock synchronization and the second section performs intra-cluster clock synchronization. The first section contains a single loop, whereas the second section is composed of two loops that are dependent upon each other (i.e., there is an inner-loop and an outer-loop). Hence, the worst-case time complexity of Algorithm 1 becomes $O(N^{2})$, where *N* refers to the number of nodes. In a similar manner, the worst-case time complexity of Algorithm 2 remains $O(N^{2})$. Since Algorithms 1 and 2 constitute the proposed PDMAC protocol, the overall worst-case complexity of PDMAC becomes $O(N^{2})$.

## 4. Performance Evaluation

This section evaluates the performance of our proposed PDMAC protocol in comparison with DMCMAC [14], CSRDS [17], and IEEE 802.11p (CSMA/CA). Simulations were performed using the VANET Toolbox [43], which is a reliable and widely used vehicular network simulator with support for MAC layer [44,45,46,47]. Table 3 lists the simulation parameters along with their configurations, which are commonly used for evaluating TDMA-based vehicular MAC protocols in the state-of-the-art [4,5,14,41]. All simulations were based on the scenario depicted in Figure 1, where the number of nodes was varied from 0 to 550, unless otherwise specified. The synchronization interval for each protocol was taken as 100 ms. Moreover, nodes were categorized into different density levels, namely, sparse, medium, and dense, in order to normalize the number of nodes in accordance with the classification of real-life traffic with respect to node density proposed in [5]. A sparse network consisted of a maximum of 200 nodes, a medium network ranged between 201 to 400 nodes, and a dense network consisted of more than 400 nodes. Performance evaluation metrics included clock synchronization, channel utilization, message loss rate, end-to-end delay, and throughput, which are commonly used in the state-of-the-art to evaluate MAC protocols in VANETs [22,33,34,48]. Analytical results, obtained on MATLAB R2018a, were used to validate the simulation results for the proposed PDMAC protocol. Each result presented was averaged over 20 replicated simulation runs by keeping all parameters fixed and changing the random seed values.

### 4.1. Clock Synchronization

**Figure 5:**
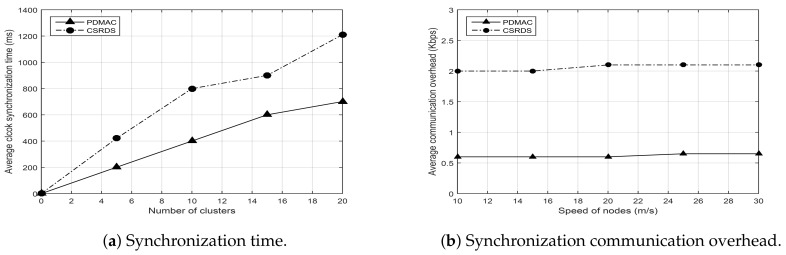
Clock synchronization.

Clock synchronization is crucial in TDMA-based protocols because time slot reservation by all nodes must occur with respect to a commonly shared clock. In a case where the clocks are unsynchronized, nodes find it difficult to reserve slots for the transmission of their messages. PDMAC addresses this issue by proposing a novel clock synchronization technique using Algorithm 1, as detailed in Section 3.2. Results depicted in Figure 5a validate the improved performance of PDMAC, where it outperformed CSRDS in terms of average synchronization time in a scenario with a variable number of clusters ranging from 0 to 20, with each cluster consisting of 5 nodes.

Moreover, we considered another scenario, with 100 nodes having variable speeds ranging from 10 to 30 m/s, to evaluate the performance of PDMAC and CSRDS for communication overhead generated during clock synchronization. Toward this end, Figure 5b presents the results where PDMAC, due to its lightweight $Sync_{CH}$ and $Sync_{MEM}$ messages, retained its superior performance.

### 4.2. Channel Utilization

**Figure 6:**
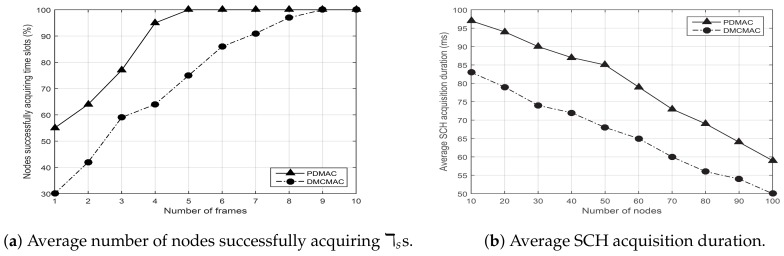
Channel utilization. DMCMAC: distributed multi-channel media access control; SCH: service channel.

The priority-based dissemination of warning messages in CCA schemes is critical. In this regard, warning messages are given higher priority during channel access. We considered a scenario with 20 nodes and variable number of frames ranging from 1 to 10. While each node transmitted warning messages, we evaluated the performance of PDMAC and DMCMAC in terms of successful time slot acquisition. Since the efficiency improved for all the protocols as the number of frames increased, similar behavior by both the protocols can be observed in the results demonstrated in Figure 6a. However, PDMAC exhibited improved performance compared to DMCMAC because of its three-tier priority-based slot reservation process.

Moreover, we considered another scenario with the number of nodes ranging from 10 to 100. As frame collisions remained proportional to the number of nodes, SCH acquisition duration also experienced performance degradation. Results depicted in Figure 6b show the same behavior for PDMAC and DMCMAC. Furthermore, due to priority-based warning messages dissemination in PDMAC, messages acquired longer SCH duration than DMCMAC, thereby providing improved warning messages delivery.

### 4.3. Message Loss Rate

**Figure 7:**
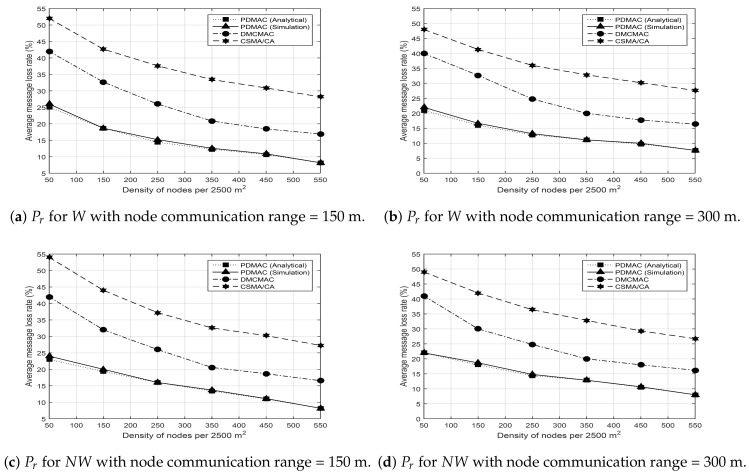
Average message loss rate. CSMA/CA: carrier-sense multiple access with collision avoidance.

The rate of message loss ( $P_{r}$) is computed as [5]

(3) $$P_{r}=\frac{\sum_{i=1}^{P_{l}}P_{l_{i}}}{P_{t}},$$

where $P_{l_{i}}$ denotes a single dropped message and $P_{t}$ represents the total number of messages transmitted across the network. The selection of $B_{f}$ is a critical decision during warning message transmission. Due to the high-speed mobility of nodes in opposite directions, frequent route changes are observed in VANETs even during the transmission of a single message, thereby producing frequent network partitions. The probability of such network partitions reduces with increasing node density because network connectivity improves with the increased number of nodes. Results shown in Figure 7a–d depict the same behavior for all the protocols.

Moreover, Figure 7a,b depicts the results of message loss rate, for warning messages only, with node communication ranges of 150 and 300 m, respectively. Here, all the protocols provided better efficiency as the communication range increased. However, the performance of PDMAC was considerably better than DMCMAC and CSMA/CA in both cases. DMCMAC exhibited better performance than CSMA/CA and provided an adaptive ${ℸ}_{s}$ reservation, where the number of ${ℸ}_{s}$ on the frames remained proportional to the number of messages. However, DMCMAC treats both warning and non-warning messages with equal priority. Here, a higher ratio of non-warning messages in the network increases the drop rate of warning messages. Conversely, the proposed PDMAC protocol employs a three-tier priority-based ${ℸ}_{s}$ reservation, which effectively reduces the drop rate of warning messages.

Furthermore, DMCMAC and CSMA/CA do not take into account the direction component during relay selection. Here, the probability of a network partition increases when a relay node bears an opposite direction to the destination node, which ultimately results in an increased message loss rate [5]. To address this, PDMAC takes into account the direction component and the distance between the relay and destination nodes in order to select a suitable relay. This ensures reliable and in-time message dissemination. The results presented in Figure 7c,d validate this claim, where PDMAC outperformed DMCMAC and CSMA/CA by achieving reduced message loss rate during the transmission of non-warning messages for nodes with communication ranges of 150 and 300 m, respectively.

### 4.4. End-to-End Delay

**Figure 8:**
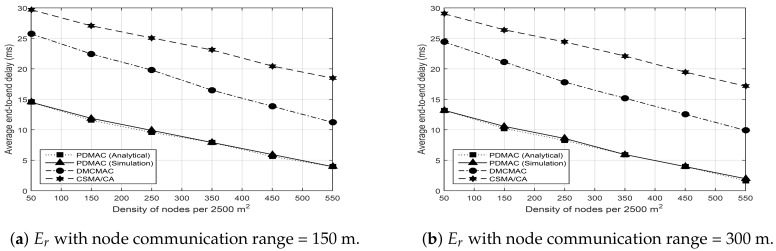
Average end-to-end delay.

This metric computes the end-to-end delay ($E_{r}$) observed during the transmission of each message as [5]

(4) $$E_{r}=\frac{\sum_{i=1}^{R_{p}}E_{d_{i}}}{R_{p}},$$

where $E_{d_{i}}$ refers to the delay experienced in transmitting an *i*th message, and $R_{p}$ represents the total number of messages successfully received at the destination. Since DMCMAC and CSMA/CA do not consider the direction component, relays selected by these protocols may bear opposite directions to the destination nodes. Due to the opposite directions of nodes, the number of relays increases on the route, which leads to an increased number of send and receive operations. As these operations are costly in terms of time during communication [49], increased end-to-end delays are experienced by DMCMAC and CSMA/CA. Furthermore, the adaptive feature of DMCMAC allocates ${ℸ}_{s}$ to all the messages. The number of ${ℸ}_{s}$ on the frame remains proportional to the number of messages. Thus, the duration of each ${ℸ}_{s}$ also increases or decreases accordingly. This implies that an increased traffic load on the network shortens the ${ℸ}_{s}$ duration on the frame in DMCMAC, which results in increased end-to-end delay. Conversely, PDMAC takes the direction component into account and the prioritized dissemination of messages to resolve the aforementioned issues. For these reasons, PDMAC outperforms DMCMAC and CSMA/CA, as shown in the results of Figure 8a,b.

### 4.5. Throughput

**Figure 9:**
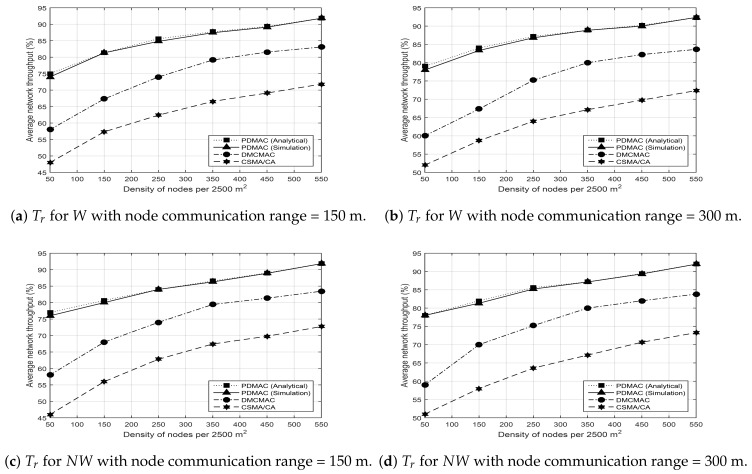
Average network throughput.

The final metric for performance evaluation refers to the achieved network throughput ($T_{r}$), which is computed as [5]

(5) $$T_{r}=\frac{\sum_{i=1}^{R_{p}}R_{p_{i}}}{P_{t}},$$

where $R_{p}{}_{i}$ symbolizes the successful reception of the *i*th message at a destination node, and $P_{t}$ represents the total number of messages originated from all source nodes. The network throughput remains inversely proportional to the message loss rate and end-to-end delay observed during message transmissions. The results presented in Figure 9a,b validate the improved throughput in the case of PDMAC, in comparison to DMCMAC and CSMA/CA, due to our novel three-tier priority assignment technique. Similarly, PDMAC also retained its superiority over DMCMAC and CSMA/CA in the results shown in Figure 9c,d.

### 4.6. Critical Discussion

In VANETs, the performance of CSMA/CA degrades with the increase in network density. Conversely, TDMA-based protocols, which divide each frame into a set of time slots to enable simultaneous message transmission, are considered more suitable for dynamic networks like VANETs. However, inefficient clock synchronization and lack of message prioritization limit the efficiency of TDMA-based protocols. So, we propose a protocol, called PDMAC, for robust inter-cluster and intra-cluster clock synchronization and better channel utilization. Furthermore, PDMAC pioneers the use of a three-tier priority-based warning message dissemination, which ensures reliability and in-time delivery.

Simulation results presented in the previous sections demonstrate the robust nature of PDMAC, which was validated further by analytical results. The results demonstrate reduced average clock synchronization time and communication overhead for PDMAC by 286 ms and 1.5 Kbps, respectively, in comparison to CSRDS. Considering channel utilization, compared with DMCMAC, PDMAC demonstrated 15% and 14% enhanced performance in successful time slot reservation and SCH acquisition duration, respectively. Moreover, for average message loss rate, end-to-end delay and network throughput, PDMAC demonstrated an improved efficiency by 11.25%, 10 ms, and 12%, respectively, over DMCMAC; and 21%, 14.96 ms, and 22%, respectively, over CSMA/CA.

The proposed PDMAC protocol can be incorporated in intelligent transportation systems to enable a safe driving environment through in-time and reliable warning messages dissemination. This will provide significant time for vehicles to adopt the communicated preventive measures, thereby minimizing road accidents. To that end, our future work will evaluate PDMAC in tandem with our previously proposed application layer protocol [4], network layer protocol [5], and secure message dissemination [2], to study their combined effect on vehicle accident prevention. The limitation of PDMAC, however, is that it is designed for warning messages dissemination on bi-directional highways and cannot cater for road intersections in urban environments. We also intend to address this limitation in our future work.

## 5. Conclusions

We proposed a cluster-based V2V MAC protocol called PDMAC for prioritized warning messages delivery in VANETs to evade road accidents on bi-directional highways. PDMAC introduces inter-cluster clock synchronization alongside intra-cluster synchronization, which leads to reduced communication overhead and improved channel utilization. Additionally, PDMAC pioneers the use of a three-tier priority assignment to ensure reliable and in-time delivery of warning messages by taking into account the direction component of nodes, message type, and severity level on each tier. Simulation and analytical results reveal that, as compared to eminent vehicular MAC protocols, PDMAC enables reduced message loss rate and end-to-end delays, and increased network throughput. Our future work includes the extension of PDMAC to cater to urban VANET environments.

## Figures and Tables

**Table 1:** List of notations.

Notation	Description
>>	Message forwarding from left to right node
ack	Acknowledgment in response to RES
$\alpha_{f}$	Set of free time slots
$B_{f}$	Best forwarder intermediary relay node
$B_{f}\text{\_}ID$	$B_{f}$ Identity
χ	Range of speeds for nodes
*C*	Set of member nodes in a cluster
$\bar{CH}$	Set of cluster heads
$CH_{B}$	A randomly selected CH for clock synchronization
$CH_{B}\text{\_}ID$	Identity of $CH_{B}$
$CH_{i}\text{\_}ID$	Identity of the ith cluster head
*D*	Destination node
${ℸ}_{s}$	Time slot on the frame to transmit messages
dec	Acceptance/rejection field of relay service
δ	Final distance between nodes
H(.)	Hamming distance function
κ	Direction component
μ	L1-norm distance between nodes
Min(.)	Minimum function
*N*	Set of all nodes
NW	A non-warning message
*R*	Set of intermediary relay nodes
Rand(.)	Random selection function
REQ	Request message
RES	Response message
ρ	Collision probability among nodes
*S*	Source node
SL	Severity level of a warning message
SN	Message type
*T*	Local clock time of a node
validate_timer	Node’s timer validation field
*W*	A warning message

**Table 2:** Severity levels of warning messages.

Severity Level (*SL*)	*SL* Value	Range of Collision Probability ($\rho_{c}$)
$SL_{0}$	00	0.00<$\rho_{c}$⩽0.33
$SL_{1}$	01	0.34<$\rho_{c}$⩽0.66
$SL_{2}$	10	0.67<$\rho_{c}$⩽1.00
NW	11	$\rho_{c}=0.00$

**Table 3:** Simulation parameters.

Parameter	Value
Simulation area	5000 m${}^{2}$
Type of road traffic	Bi-directional highway
Cluster size	Variable
Speed of nodes	0–42 m/s
Regular acceleration, deceleration	1–6 m/s${}^{2}$
Number of nodes	0–550
Transmission range	150 m, 300 m
Number of channels	1 control channel (CCH) and 6 SCH
Synchronization interval	100 ms
Data transmission rate	12 Mbps
Simulation time	300 s
